# Captive lizards (Squamata: Sauria) as reservoirs of medically important and antifungal-resistant yeasts

**DOI:** 10.1093/mmy/myag083

**Published:** 2026-08-17

**Authors:** Rossella Samarelli, Nicola Pugliese, Jairo Alfonso Mendoza-Roldan, Mara Miglianti, Renata Fagundes-Moreira, Pietro Laricchiuta, Matteo Legrottaglie, Patrizia Danesi, Domenico Otranto, Gustavo Giusiano

**Affiliations:** Department of Veterinary Medicine, University of Bari “Aldo Moro”, Valenzano, 70010 Bari, Italy; Department of Veterinary Medicine, University of Bari “Aldo Moro”, Valenzano, 70010 Bari, Italy; Department of Veterinary Medicine, University of Bari “Aldo Moro”, Valenzano, 70010 Bari, Italy; Department of Veterinary Medicine, University of Bari “Aldo Moro”, Valenzano, 70010 Bari, Italy; Department of Veterinary Medicine, University of Bari “Aldo Moro”, Valenzano, 70010 Bari, Italy; National PhD Program in One Health Approaches to Infectious Diseases and Life Science Research, Department of Public Health, Experimental and Forensic Medicine, University of Pavia, 27100, Pavia, Italy; Zoo Safari di Fasano, 72015, Brindisi, Italy; Zoo Safari di Fasano, 72015, Brindisi, Italy; Istituto Zooprofilattico Sperimentale delle Venezie, 35020, Legnaro, PD, Italy; Department of Veterinary Medicine, University of Bari “Aldo Moro”, Valenzano, 70010 Bari, Italy; Department of Veterinary Clinical Sciences, City University of Hong Kong, 99077, Hong Kong Special Administrative Region; Department of Veterinary Medicine, University of Bari “Aldo Moro”, Valenzano, 70010 Bari, Italy; Departamento de Micología, Instituto de Medicina Regional, Universidad Nacional del Nordeste, CONICET, 3500, Resistencia, Argentina

**Keywords:** captive lizards, reservoir, yeasts, opportunistic fungi, antifungal resistance, one health

## Abstract

Zoological collections represent unique environments in which captivity-related alterations of host microbiota may increase susceptibility to infectious diseases. While reptiles are recognized reservoirs of microorganisms, data on quantitative yeast colonization and antifungal susceptibility in captive lizards remain limited. This study assessed 28 lizards belonging to nine species housed in a zoological park in southern Italy. Skin and cloacal swabs (*n* = 56) revealed yeast growth in 75% of animals and 57.1% of samples, with positivity rates of 60.7% and 53.6% for skin and cloacal samples, respectively. MALDI-TOF MS and ITS region sequencing identified 113 isolates belonging to 17 distinct fungal species, including *Arthrographis* spp.*, Meyerozyma* spp.*, Candida* spp.*, Pichia* spp.*, Trichosporon* spp., and others. *Arthrographis kalrae* was the predominant species (i.e., 36.3%), followed by *Meyerozyma guilliermondii* (i.e., 16.8%). Mean colonization loads were 4.25 CFU/swab for skin and 10.50 CFU/swab for cloacal samples. Eight species were considered clinically relevant, including the *Candida parapsilosis* complex, *Pichia kudriavzevii*, and *Trichosporon asahii*. Antifungal susceptibility testing performed according to Clinical and Laboratory Standards Institute (CLSI, 2022) guidelines revealed marked phenotypic heterogeneity and frequent elevation of minimum inhibitory concentration (MIC). Above-threshold MICs differed significantly according to yeast species, antifungal compound, and sampling site. Non-wild-type phenotypes and elevated MICs were observed across multiple antifungal classes, with amphotericin B >64 µg/ml and fluconazole >256 µg/ml in several isolates (i.e., 92.3% and 75.0%, respectively). Reduced susceptibility or resistance was evident in *Clavispora lusitaniae, M. guilliermondii*, and *Wickerhamomyces anomalus*. These findings support the hypothesis that captive lizards may represent asymptomatic reservoirs of opportunistic and potentially antifungal-resistant yeasts, emphasizing the importance of hygiene measures and microbiological surveillance in zoological settings.

## Introduction

Zoological collections play an essential role in biodiversity conservation, education, and scientific research. However, captivity imposes artificial environmental conditions (e.g., restricted movement, controlled microclimates, modified diets, and close animal-human interaction), which significantly alter the host microbiota and increase susceptibility to infectious diseases and zoonotic transmission ^1^ In reptiles, these alterations may affect both bacterial and fungal communities, with consequences for animal and public health. ^2,3^ Previous studies have reported yeasts colonizing skin, mucosal membranes, and gastrointestinal tracts of reptiles, suggesting that captive animals may act as reservoirs of opportunistic and potentially pathogenic fungi. ^4,5^

Some yeast genera, including *Candida, Geotrichum*, and *Malassezia*, are considered part of the normal reptile microbiota.^5^ However, stress, immunosuppression, poor husbandry, or environmental imbalance may favor opportunistic behavior, conditions frequently associated with captivity. ^6^ For instance, the emerging pathogen *Candidozyma auris* (syn. *Candida auris*), recognized as one of the most urgent fungal threats to public health, was isolated from the cloaca of an Egyptian cobra (*Naja haje legionis*), suggesting the potential role of reptiles as reservoirs of this species. ^7^ Consequently, reptile-associated yeasts may contribute to animal disease and represent a potential source of zoonotic exposure, particularly in settings characterized by frequent human–animal contact, such as zoological facilities and cultural rituals. ^5,8^

These concerns are intensified by the global emergence of antifungal resistance, now recognized as a major challenge in both human and veterinary medicine. Reflecting this threat, the World Health Organization (WHO) has included several fungal species in its priority pathogens list, highlighting the need for surveillance across clinical, veterinary, and environmental settings. ^9,10^ Resistance to azoles and echinocandins has increasingly been reported in clinically relevant yeasts such as *Candida albicans, C. auris*, and *Cryptococcus* spp., compromising therapeutic efficacy and contributing to poor clinical outcomes. ^9,11^ In zoological settings, empirical antifungal treatments and environmental exposure to antifungal residues may favor the persistence and circulation of resistant yeasts, potentially facilitating interspecies transmission within a One Health context. ^12^

Although reptiles may act as reservoirs of antifungal-resistant yeasts, studies specifically focused on captive lizards remain scarce, particularly regarding quantitative colonization patterns and antifungal susceptibility profiles. Moreover, their epidemiological role in zoological facilities and contribution to the maintenance and spread of antifungal-resistant yeasts have not yet been systematically investigated. Therefore, comprehensive characterization of fungal communities colonizing captive lizards, including taxonomic identification, quantitative fungal burden assessment, and antifungal susceptibility profiling, is essential to better understand their potential role as reservoirs of medically relevant yeasts and the associated zoonotic implications within a One Health perspective.

## Materials and methods

### Lizards sampling and husbandry

As part of a survey investigating parasitic agents in reptiles at the Zoosafari Fasano (Fasano, Southern Italy), 28 lizards belonging to nine species (Table 1) were sampled for yeast detection. All reptiles were housed in the zoo reptile facility, a climate-controlled environment composed of multiple enclosures containing one or more individuals of the same species. Enclosures included environmental enrichment elements, such as vegetation and artificial shelters. Animals were fed with fresh vegetables, thawed prey, or live insects according to their dietary needs. Access to enclosures was restricted to authorized keepers for feeding, cleaning, and routine management.

**Table 1:** Quantitative yeast colonization in captive lizards based on skin and cloacal swab cultures. Colony-forming unit (CFU) counts are reported for each anatomical site, together with the total fungal burden per animal and cumulative CFU counts for each host species.

ID Lizard	Species	Skin CFU	Cloacal CFU	Total CFU per animal	Total CFU per specie
L001	*Broadleysaurus major*	9	0	9	>262*
L002	*Broadleysaurus major*	4	3	7	
L003	*Broadleysaurus major*	6	18	24	
L004	*Broadleysaurus major*	9	>200	>209	
L005	*Broadleysaurus major*	3	10	13	
L006	*Laudakia stellio*	0	0	0	3
L007	*Laudakia stellio*	1	0	1	
L008	*Laudakia stellio*	0	0	0	
L009	*Laudakia stellio*	1	1	2	
L010	*Salvator merianae*	0	0	0	0
L011	*Tupinambis rufescens*	0	0	0	57
L012	*Tupinambis rufescens*	55	2	57	
L013	*Salvator merianae*	0	0	0	0
L014	*Cyclura cornuta*	0	2	2	30
L015	*Cyclura cornuta*	0	1	1	
L016	*Cyclura cornuta*	1	7	8	
L017	*Cyclura cornuta*	0	0	0	
L018	*Cyclura cornuta*	0	19	19	
L019	*Iguana iguana*	8	0	8	45
L020	*Iguana iguana*	2	0	2	
L021	*Iguana iguana*	3	1	4	
L022	*Iguana iguana*	10	2	12	
L023	*Iguana iguana*	2	17	19	
L024	*Iguana iguana*	0	0	0	
L025	*Varanus cumingi*	1	0	1	1
L026	*Tiliqua gigas*	1	10	11	12
L027	*Tiliqua gigas*	0	1	1	1
L028	*Hydrosaurus amboinensis*	3	0	3	3
**Totals**	**Species *n* = 9**	**119**	**>294**	**> 413**	**>413**

* Values expressed as ‘‘>’’ indicate counts exceeding the upper quantification limit of the culture method.

### Sample collection, culture methods, biochemical tests, and MALDI-TOF MS identification

All reptiles underwent skin swabbing along the dorsal and ventral surfaces and cloacal swabbing. To minimize contamination and handling-related transmission, yeast sampling was performed before any other procedures. Gloves were changed between animals, and all procedures were conducted using swabs moistened with sterile saline solution, placed in sterile transport tubes, refrigerated, and processed upon arrival at the laboratory.

Samples were cultured on three media: Sabouraud dextrose agar supplemented with chloramphenicol (0.5 g/l; SDA, BioLife ®, Milan, Italy), incubated at 25°C and at 32°C for 7 days; Fast Fung agar, ^13^ incubated at 32°C for 7 days; and selective *C. auris* medium, ^14^ incubated at 42°C for 14 days. Plates were observed daily for fungal growth. Cultures were considered positive after microscopic confirmation by Gram staining. Colony counts were recorded and expressed as CFUs.

For each positive sample, at least four morphologically distinct colonies, based on color, size, and texture, were subcultured for further characterization. Isolates underwent urease and germ tube testing. Final identification was based on colony morphology, microscopic examination, biochemical profiling, and mass spectrometry Biotyper MALDI-TOF MS (Bruker Daltonics, Bremen, Germany). ^15^

### Molecular identification

Molecular identification was performed for isolates yielding inconclusive MALDI-TOF MS results (score < 1.70) or identification restricted to the genus level. Genomic DNA was extracted using the DNeasy Blood & Tissue Kit (QIAGEN, Hilden, Germany), following the manufacturer’s instructions. The internal transcribed spacer (ITS) region was amplified using ITS1 (5′ -TCCGTAGGTGAACCTGCGG-3′) and ITS4 (5′ - TCCTCCGCTTATTGATATGC-3′) primers. PCR (Polymerase Chain Reaction) products were purified and sequenced in both directions using BigDye Terminator v.3.1 chemistry in an automated sequencer (ABI-PRISM 377). Sequence analyses were conducted using the CLC Genomic Workbench v. 22.0.1 (Qiagen Digital Insight, Aarhus, Denmark). Forward and reverse reads were assembled using CAP3 software, ^16^ and consensus sequences were compared against GenBank using the Basic Local Alignment Search Tool (BLAST) ^17^ and the Mycobank database (https://www.mycobank.org).

### Antifungal susceptibility testing

All isolates were subjected to antifungal susceptibility testing using the Clinical and Laboratory Standards Institute (CLSI) broth microdilution method (M27-M44S) for determination of minimum inhibitory concentration (MIC). ^18^ Clinical breakpoints (CBPs) were interpreted according to the CLSI M27-M44S document, whereas epidemiological cutoff values (ECVs) were interpreted following CLSI M57S recommendations. ^19^ The antifungal panel included representatives of the major antifungal classes: fluconazole (FCZ, 0.125–256 μg/ml), itraconazole (ITZ, 0.016–32 μg/ml), voriconazole (VOR, 0.016–32 μg/ml), and posaconazole (PSZ, 0.016–32 μg/ml), amphotericin B (AMB, 0.03–64 μg/ml), and anidulafungin (AND, 0.016–32 μg/ml).

### Statistical analysis

Isolation frequency data were analyzed as paired observations. Differences in yeast detection frequency between skin and cloacal swabs were evaluated using an exact McNemar test. Differences in paired CFU loads between skin and cloacal samples were assessed using the Wilcoxon signed-rank test. In addition, a sign test was used to evaluate the direction of paired differences between the two anatomical sites.

To analyze species/drug combinations for which ECVs or CBPs were available, isolates with MIC values at or below the selected CBP were classified as susceptible, and the others as non-susceptible. Similarly, isolates with MIC values at or below the ECVs were classified as wild-type (WT); otherwise, they were classified as non-wild-type (NWT). The MIC values greater than the highest tested concentration were treated as right-censored values and considered above the cut-off.

Associations among cut-off classification, species, antifungal compound, and sampling site were evaluated using Fisher’s exact test. For contingency tables larger than 2 × 2, the test was performed by using Monte Carlo simulation with 10 000 replicates, and pairwise Fisher’s exact tests were applied as post-hoc analyses. The *P*-values from pairwise comparisons were adjusted for multiple testing using the Benjamini–Hochberg (BH) false discovery rate. The MIC distributions were evaluated for all the isolates to compare antifungal activity patterns among species, and MIC_50_ and MIC_90_ were calculated when the sample size consisted of at least 5 or 10 isolates, respectively. The MIC values were analyzed on a log_2_ scale, and model-estimated MICs and 95% confidence intervals (CIs) were obtained from the censored regression models and back-transformed from the log₂ scale. To compare MIC distributions among species, drug-specific censored Tobit-like regression models were fitted using log₂ of MIC values as a dependent variable and species as an explanatory variable. Only species represented by at least five isolates were included. The species effect was evaluated separately for each antifungal by using a likelihood-ratio comparison between the species model and the corresponding null model.

For antifungals showing a significant species effect, pairwise post-hoc comparisons among species were performed by using the BH false discovery rate. Statistical significance was set at *P* < 0.05. All the analyses were performed in R v. 4.6.0. ^20^

## Results

### Yeast colonization and fungal burden

A total of 56 skin and cloacal swabs were collected from 28 lizards representing nine species (Table 1). Yeast growth obtained at 32°C was detected in 32/56 (i.e., 57.14%) samples, corresponding to 21/28 (i.e., 75%) positive animals. Skin and cloacal positivity rates were 60.7% (*n* = 17/28) and 53.6% (*n* = 15/28), respectively. Among colonized lizards, 28.6% (*n* = 6/21) showed exclusive skin colonization, 19.0% (*n* = 4/21) exclusive cloacal colonization, and 52.4% (*n* = 11/21) simultaneous colonization in both sites.

Although filamentous fungi were occasionally recovered during culture at 25°C, these isolates were excluded from further analysis as the focus of this study was characterizing the yeast microbiota.

No significant difference in yeast detection frequency was observed between skin and cloacal samples (*P* = 0.754). Quantitative analysis revealed mean colonization loads of 4.25 CFU/swab for skin and 10.50 CFU/swab for cloacal samples, with counts ranging from 1 to >200. Values reported as >200 were treated as minimum estimates for the statistical analysis. Although cloacal samples showed higher mean CFU burden, differences between sites were not significant (*P* = 0.600). Similarly, the direction of paired differences was balanced, with higher cloacal loads in ten animals, higher skin loads in ten animals, and equivalent loads in eight animals (*P* = 1.000). Among host species, *Broadleysaurus major* showed the highest yeast burden (>262 colonies out of >413 total recovered), whereas no yeasts were isolated from *Salvator merianae* (Table 1).

### Species diversity and distribution

A total of 113 yeast isolates were recovered, including 62 from skin and 51 from cloacal samples. Identification by biochemical tests, MALDI-TOF MS, and ITS region sequencing revealed 17 distinct species. Species distribution and anatomical localization are summarized in Table 2. *Arthrographis kalrae* was the predominant species overall, followed by *M. guilliermondii* and *Meyerozyma caribbica*. Skin samples were mainly dominated by *A. kalrae* and *M. guilliermondii*, whereas cloacal samples showed a broader species diversity, including several taxa exclusively or preferentially detected at this site.

**Table 2:** Yeast species isolated from skin and cloacal swabs of captive lizards, including anatomical distribution of isolates and antifungal susceptibility profiles.

Species	N. Isolates/Skin (%)	N. Isolates/Cloaca (%)	N. tot (%)	Interpretation criteria	FCZ^1^	ITZ^2^	VOR^3^	PSZ^4^	AMB^5^	AND^6^
*Arthrographis kalrae*	30 (48.4%)	11 (21.6%)	41 (36.3%)	MIC-based	4–>256	0.5–>32	0.125– >32	0.5–32	2–>64	1–>32
*Blastobotrys chiropterorum*	0	2 (3.9%)	2 (1.8%)	MIC-based	256	> 32	8	8	16	8
*Blastobotrys raffinosifermentans*	1 (1.6%)	0	1 (0.9%)	MIC-based	> 256	> 32	4	> 32	4	8
*Candida intermedia*	1 (1.6%)	0	1 (0.9%)	MIC-based	128	2	0.25	1	4	2
*Candida orthopsilosis*	2 (3.2%)	0	2 (1.8%)	ECV	16	1	0.125	1	8	2
*Candida palmioleophila*	0	6 (11.8%)	6 (5.3%)	MIC-based	32	8	0.25	2	8	4
*Candida parapsilosis*	1 (1.6%)	5 (9.8%)	6 (5.3%)	CBP/ECV	8–128	1–> 32	0.03–0.5	0.5–32	8	2–4
*Clavispora lusitaniae*	4 (6.4%)	1 (1.9%)	5 (4.4%)	MIC-based/ECV	1–128	1–> 32	0.03–> 32	0.5–4	4–16	1–4
*Geotrichum candidum*	0	1 (1.9%)	1 (0.9%)	MIC-based	64	> 32	16	16	4	> 32
*Meyerozyma caribbica*	4 (6.4%)	5 (9.8%)	9 (8.0%)	MIC-based	8 – > 256	8–>32	0.125 – >32	2–32	8–>64	4–> 32
*Meyerozyma guilliermondii*	18 (29.0%)	1 (1.9%)	19 (16.8%)	MIC-based/CBP/ECV	4–>256	1–> 32	0.125 – >32	0.5–32	2–>64	2–32
*Pichia fermentans*	0	2 (3.9%)	2 (1.8%)	MIC-based	>256	>32	>32	32	>64	> 32
*Pichia kluyveri*	0	4 (7.8%)	4 (3.5%)	MIC-based	128–>256	2–>32	1–4	2–4	2–4	0.125
*Pichia kudriavzevii*	0	1 (1.9%)	1 (0.9%)	CBP/ECV	R	8	0.5	2	8	16
*Torulaspora delbrueckii*	0	4 (7.8%)	4 (3.5%)	MIC-based	1 – >256	2–>32	0.03–>32	0.5–32	4–>64	2–32
*Trichosporon asahii*	1 (1.6%)	5 (9.8%)	6 (5.3%)	MIC-based	1 – > 256	2–> 32	0.03–> 32	0.5–>32	4–>64	R*
*Wickerhamomyces anomalus*	0	3 (5.9%)	3 (2.6%)	MIC-based/ECV	16–>256	4–>32	0.5–>32	4–32	8–>64	0.25–32
Tot. 17	Tot. 62	Tot. 51	Tot. 113							

Note: MIC ranges (µg/ml) are reported for each antifungal agent tested. Interpretation of susceptibility profiles was based on CLSI, CBPs, and/or ECVs, when available; otherwise, interpretation relied on MIC distribution analysis.

Abbreviations: FCZ^1^, fluconazole; ITZ^2^, itraconazole; VOR^3^, voriconazole; PSZ^4^, posaconazole; AMB^5^, amphotericin B; AND^6^, anidulafungin.

* R indicates intrinsic resistance to the corresponding antifungal agent.

### Antifungal susceptibility profiles

Antifungal susceptibility testing revealed marked phenotypic heterogeneity and frequently elevated MIC values across isolates. Overall MICs ranged from 1 to >256 µg/ml for FCZ, 0.5 to >32 µg/ml for ITZ, 0.03 to >32 µg/ml for VOR, 0.5 to >32 µg/ml for PSZ, 0.125 to >32 µg/ml for AND, and 2 to >64 µg/ml for AMB (Table 2).

For species with CLSI CBPs, most isolates of the *Candida parapsilosis* complex remained susceptible to azoles, although a subset showed resistance to FCZ, with all isolates exhibiting elevated AMB MICs (i.e., 8 µg/ml) while remaining susceptible to AND. *Candida orthopsilosis* isolates presented VOR MICs at the upper epidemiological cutoff limit, suggesting reduced susceptibility. Among *M. guilliermondii*, resistance to AND was detected in 2/19 isolates. *Pichia kudriavzevii* remained susceptible to VOR; FCZ susceptibility was not evaluated due to intrinsic resistance according to CLSI guidelines.

For species lacking CBPs, ECVs were applied to distinguish WT from NWT phenotypes. Elevated frequencies of NWT isolates were detected across several species and antifungal classes. NWT phenotypes were observed for AMB in the *C. parapsilosis* complex, *Clavispora lusitaniae, M. guilliermondii* and *P. kudriavzevii*; for AND in *C. lusitaniae*; for FCZ in *M. guilliermondii, C. lusitaniae*, and *C. orthopsilosis*; for ITZ in *C. lusitaniae, C. parapsilosis* complex*, M. guilliermondii*, and *P. kudriavzevii*, and for PSZ in *C. lusitaniae* and *M. guilliermondii*.

**Figure 1:**
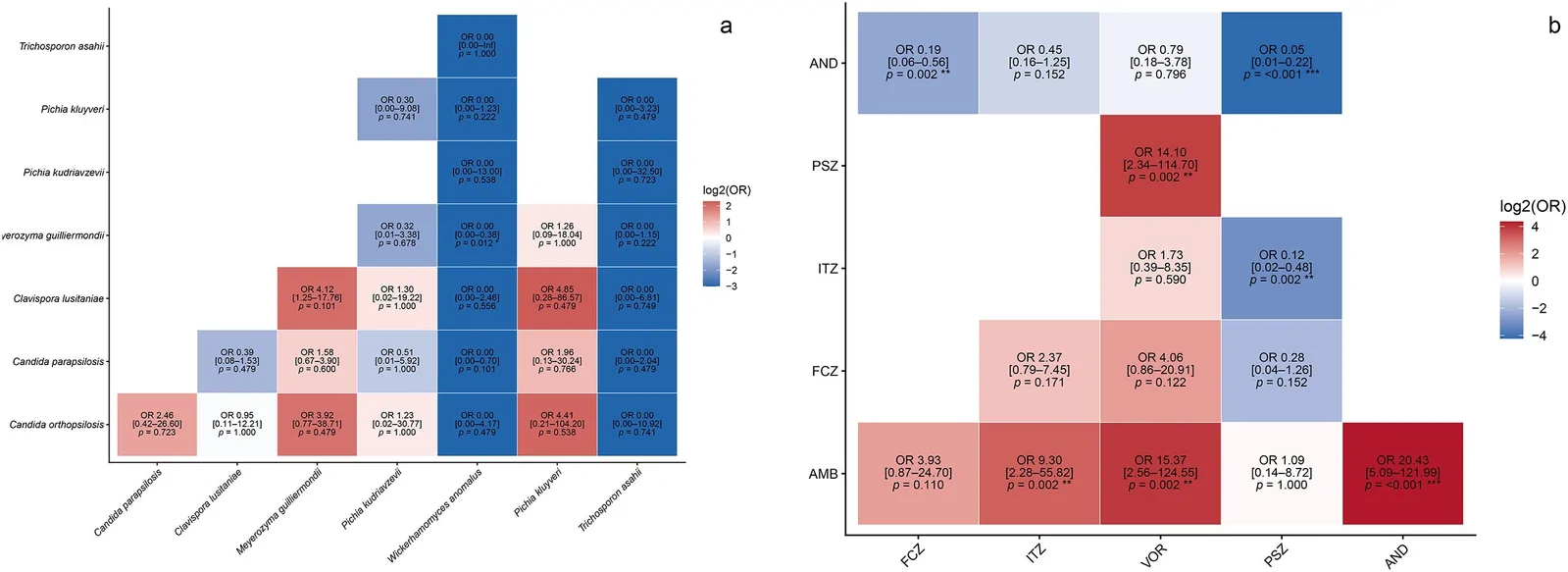
Heatmap of pairwise comparison of the proportions of MICs above thresholds among the yeast species (a) and considering the antifungal drugs (b). Asterisks indicate statistical significance after BH correction for multiple pairwise comparisons: *P* < 0.05: *; *P* < 0.01: **; *P* < 0.001: ***. AMB: amphotericin B; AND: anidulafungin; FCZ: fluconazole; ITZ: itraconazole, PSZ: posaconazole; VOR: voriconazole.

Considering all interpretative thresholds, including CBPs, ECVs, and intrinsic resistance profiles, 135/198 (i.e., 68.2%) isolate-drug combinations showed MIC values above the respective thresholds. The frequency of above-threshold MICs differed significantly among species and antifungal compounds (*P* = 0.001; Table 3). The highest proportions of above-threshold MICs were observed for AMB (*n =* 36/39, 92.3%) and PSZ (*n =* 33/36, 91.7%), followed by FCZ (*n =* 27/36, 75.0%) and ITZ (*n =* 20/36, 55.6%). Lower rates were recorded for VOR (*n =* 5/12, 41.7%) and AND (*n =* 14/39, 35.9%). While the significance of Fisher’s exact test at the species level, adjusted pairwise comparisons identified only one significant contrast, specifically between *M. guilliermondii* and *Wickerhamomyces anomalus* (Fig. 1a). In this case, *W. anomalus* showed a higher proportion of above-threshold MICs than *M. guilliermondii* but with a marginal OR (OR = 0.00, 95% CI: 0.00–0.38; adjusted *P* = 0.012). *Post-hoc* analyses confirmed AMB and PSZ as the compounds most frequently associated with elevated MICs (Fig. 1b). Among the remaining comparisons, only FCZ versus AND showed a significant difference (OR = 5.23, 95% CI: 1.78–16.55; adjusted *P* = 0.002).

**Table 3:** Distribution of isolates showing MIC values above the applicable interpretive threshold (AT) by yeast species and antifungal agent. Only species-drug combinations for which cutoff values (ECVs) were available are included.

Species	FCZ^1^ AT/total (%)	ITZ^2^ AT/total (%)	VOR^3^ AT/total (%)	PSZ^4^ AT/total (%)	AND^5^ AT/total (%)	AMB^6^ AT/total (%)	Overall AT/total (%)
*Candida orthopsilosis*	2/2 (100%)*	2/2 (100%)*	2/2 (100%)*	2/2 (100%)*	0/2 (0%)*	2/2 (100%)*	10/12 (83.3%)
*Candida parapsilosis*	6/6 (100%)	6/6 (100%)*	0/6 (0%)	6/6 (100%)*	0/6 (0%)	6/6 (100%)*	24/36 (66.7%)
*Clavispora lusitaniae*	4/5 (80%)*	2/5 (40%)*	–	5/5 (100%)*	Intrinsic	Intrinsic	21/25 (84%)
*Meyerozyma guilliermondii*	11/19 (57.9%)*	6/19 (31.6%)*	–	16/19 (84.2%)*	2/19 (10.5%)	18/19 (94.7%)*	53/95 (55.8%)
*Pichia kudriavzevii*	Intrinsic#	1/1 (100%)*	0/1 (0%)	1/1 (100%)*	1/1 (100%)	–	4/5 (80%)
*Wickerhamomyces anomalus*	3/3 (100%)*	3/3 (100%)*	3/3 (100%)*	3/3 (100%)*	–	3/3 (100%)*	15/15 (100%)
*Pichia kluyveri*	–	–	–	–	–	2/4 (50%)*	2/4 (50%)
*Trichosporon asahii*	–	–	–	–	6/6 (100%)*	–	6/6 (100%)

* ECV-based classification; #Recognized intrinsic resistance for the combination, with all strains considered as above-threshold.

FCZ^1^, fluconazole; ITZ^2^, itraconazole; VOR^3^, voriconazole; PSZ^4^, posaconazole; AND^5^, anidulafungin; AMB^6^, amphotericin B.

Isolates recovered from cloacal samples showed significantly higher frequencies of above-threshold MICs than those from skin samples (*P* = 0.010; OR = 2.40, 95% CI: 1.16–5.17). MIC distributions also differed significantly among species for all antifungal compounds tested (AMB: *P* = 0.001; AND: *P* < 0.001; FCZ: *P* = 0.002; ITZ: *P* < 0.001; PSZ: *P* = 0.003; VOR: *P* < 0.001).

**Figure 2:**
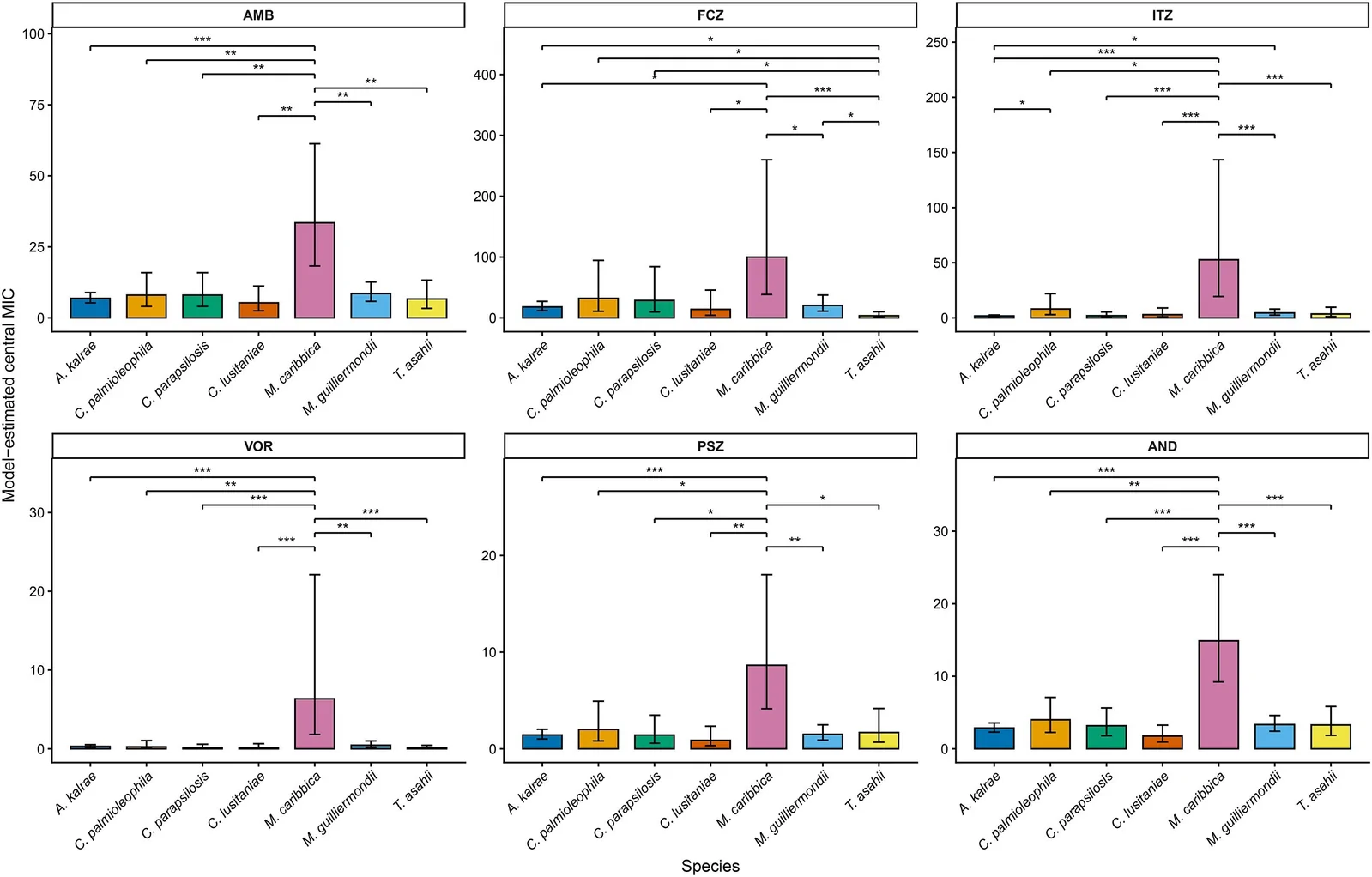
Bar plots showing the model-estimated central MIC values for the six antifungal compounds for the species whose sample size was large enough to calculate values. Significance levels are *P* < 0.05: *; *P* < 0.01: **, *P* < 0.001: ***. AMB: amphotericin B; AND: anidulafungin; FCZ: fluconazole; ITZ: itraconazole, PSZ: posaconazole; VOR: voriconazole.

The most prevalent species, *A. kalrae* (*n* = 41), showed broad phenotypic variability, particularly for FCZ, with MICs ranging from 4 to > 256 µg/ml, indicating heterogeneous susceptibility profile. *Meyerozyma caribbica*, exhibited the highest model-estimated central MICs for all antifungal compounds tested (Table 4, Fig. 2), with significantly higher MIC distributions than most other species. The largest interspecies differences were observed for ITZ and VOR. For ITZ, *M. caribbica* showed an estimated central MIC of 52.69 µg/ml (95% CI: 19.36–143.40), whereas estimates for the remaining species ranged from 1.74 µg/ml in *A. kalrae* to 8.00 µg/ml in *Candida palmioleophila*. Similarly, for VOR, *M. caribbica* exhibited the highest estimated central MIC (6.35 µg/ml; 95% CI: 1.82–22.11), while most remaining species showed estimated values below 0.5 µg/ml.

**Table 4:** Antifungal susceptibility profiles of the most representative yeast species isolated from captive lizards. MIC data are presented as MIC_50_, MIC_90_, and model-estimated central MIC values with corresponding 95% confidence intervals for each antifungal agent tested.

Species		AMB^1^		FCZ^2^		ITZ^3^		VOR^4^		PSZ^5^		AND^6^	
	n	MIC_50_/MIC_90_	Est. MIC* (95% CI)	MIC_50_/MIC_90_	Est. MIC (95% CI)	MIC_50_/MIC_90_	Est. MIC (95% CI)	MIC_50_/MIC_90_	Est. MIC (95% CI)	MIC_50_/MIC_90_	Est. MIC (95% CI)	MIC_50_/MIC_90_	Est. MIC (95% CI)
*Artrographis kalrae*	41	8/8	6.85 (5.26–8.91)	16/64	17.85 (11.78–27.06)	1/4	1.74 (1.18–2.57)	0.25/0.25	0.3 (0.17–0.52)	1/4	1.43 (1.01–2.01)	2/4	2.86 (2.29–3.56)
*Blastobotrys chiropterorum*	2	—	—	—	—	—	—	—	—	—	—	—	—
*Blastobotrys raffinosifermentans*	1	—	—	—	—	—	—	—	—	—	—	—	—
*Candida intermedia*	1	—	—	—	—	—	—	—	—	—	—	—	—
*Candida orthopsilosis*	2	—	—	—	—	—	—	—	—	—	—	—	—
*Candida palmioleophila*	6	8/—	8 (4.02–15.91)	32/—	32 (10.82–94.63)	8/—	8 (2.92–21.94)	0.25/—	0.25 (0.06–1.04)	2/—	2 (0.81–4.92)	4/—	4 (2.26–7.09)
*Candida parapsilosis*	6	8/—	8 (4.02–15.91)	16/—	28.51 (9.64–84.3)	1/—	1.92 (0.69–5.3)	0.25/—	0.14 (0.03–0.58)	1/—	1.41 (0.57–3.48)	4/—	3.17 (1.79–5.63)
*Clavispora lusitaniae*	5	4/—	5.28 (2.48–11.21)	8/—	13.93 (4.25–45.68)	1/—	2.91 (0.95–8.9)	0.03/—	0.13 (0.03–0.65)	0.5/—	0.87 (0.32–2.33)	1/—	1.74 (0.93–3.26)
*Geotrichum candidum*	1	—	—	—	—	—	—	—	—	—	—	—	—
*Meyerozyma caribbica*	9	>64/—	33.47 (18.28–61.28)	>256/—	99.92 (38.4–259.99)	>32/—	52.69 (19.36–143.4)	>32/—	6.35 (1.82–22.11)	16/—	8.64 (4.14–18.02)	16/—	14.88 (9.22–24)
*Meyerozyma guilliermondii*	19	8/>64	8.55 (5.8–12.62)	16/>256	20.3 (10.99–37.48)	2/>32	4.45 (2.49–7.94)	0.25/16	0.45 (0.2–1)	1/8	1.49 (0.9–2.48)	4/8	3.33 (2.42–4.6)
*Pichia fermentans*	2	—	—	—	—	—	—	—	—	—	—	—	—
*Pichia kluyveri*	4	—	—	—	—	—	—	—	—	—	—	—	—
*Pichia kudriavzevii*	1	—	—	—	—	—	—	—	—	—	—	—	—
*Torulaspora delbrueckii*	4	—	—	—	—	—	—	—	—	—	—	—	—
*Trichosporon asahii*	6	4/—	6.64 (3.32–13.27)	1/—	3.37 (1.13–10.04)	2/—	3.45 (1.25–9.58)	0.03/—	0.1 (0.02–0.44)	0.5/—	1.68 (0.68–4.17)	2/—	3.28 (1.84–5.83)
*Wickerhamomyces anomalus*	3	—	—	—	—	—	—	—	—	—	—	—	—

AMB^1^: amphotericin B; FCZ^2^: fluconazole; ITZ^3^: itraconazole; VOR^4^, voriconazole; PSZ^5^: posaconazole. AND^6^: anidulafungin. *Est. MIC: model-estimated central value of MIC; 95% CI: 95% confidence interval; —: Not enough data for calculating values.

*Wickerhamomyces anomalus* exhibited elevated MIC profiles, including FCZ MICs >256 µg/ml and AMB MICs reaching 64 µg/ml in several isolates. *Trichosporon asahii* showed relatively homogeneous susceptibility patterns, although one isolate presented elevated MICs for all antifungal agents tested. Other clinically relevant species, including the *C. parapsilosis* complex and *P. kudriavzevii*, displayed variable susceptibility profiles. In contrast, species such as *Candida intermedia, Pichia kluyveri, Pichia fermentans, Geotrichum candidum*, and *Torulaspora delbrueckii* showed heterogeneous MIC distributions without consistent patterns suggestive of reduced susceptibility.

## Discussion

The high prevalence and diversity of yeasts observed in this study support the hypothesis that captive reptiles housed in zoological facilities may act as important reservoirs of opportunistic fungal species. Compared with wild lizard populations, in which fungal colonization and mycobiota diversity are generally lower, ^21,22^ captive conditions may favor the establishment of more stable and diverse yeast communities; indeed, baseline data from free-living synanthropic lizards in Southern Europe (such as *Podarcis siculus, Chalcides ocellatus*, and *Tarentola mauritanica*) demonstrate that wild populations can act as reservoirs for potentially pathogenic zoonotic yeasts, primarily *Candida albicans* (44%), *Trichosporon coremiiforme* (12.1%), *Pichia kudriavzevii* (8.8%), and *T. asahii* (7.7%). ^8^

Our results clearly reflect this captivity-driven diversification; specifically, the identification of 17 yeast species, including several opportunistic pathogens, reflects the growing shift from classical pathogens toward emerging and uncommon yeast increasingly reported in both clinical and environmental settings. ^23,24^ A major finding of this study was the predominance of *A. kalrae*, which represented 36.3% (*n* = 41/113) of all isolates and was mainly recovered from skin samples, followed by *M. guilliermondii* with 16.8% (*n* = 19/113). Although traditionally considered an environmental saprophyte, *A. kalrae* has increasingly been reported as an opportunistic pathogen in both human and veterinary medicine, affecting immunosuppressed hosts. ^25–27^ Its repeated isolation from clinically healthy lizards suggests that this species may represent a stable component of the reptile skin mycobiota while simultaneously acting as a potential source of human environmental exposure for zoo handlers and veterinarians in frequent contact with these animals. Additionally, *Meyerozyma* complex, including *M. caribbica*, is also of clinical interest due to its association with emerging antifungal resistance in hospital settings. ^28^

The predominance of *Candida* and related genera align with previous studies in reptiles and captive wildlife, where these yeasts are frequently associated with mucosal and gastrointestinal niches. ^8,29^

Conversely, although genera of yeasts such as *Malassezia* are recognized as common components of the reptile microbiota,^5^ no isolates were recovered in the present study. Notably, this absence was observed despite the use of Fast Fung medium, which supports the growth of lipophilic and fastidious fungi, suggesting that the lack of *Malassezia* isolation reflects a true biological characteristic of the sampled population rather than a methodological limitation. Although often considered commensals, several of these species may behave as opportunistic pathogens, particularly in immunocompromised conditions. ^30,31^ In the present study, the detection of species such as *M. guilliermondii, C. lusitaniae, W. anomalus, P. kudriavzevii*, and *T. asahii* is noteworthy because these yeasts are increasingly implicated with healthcare-associated infections and emerging antifungal resistance worldwide, ^32,33^ confirming the global epidemiological trend of non-*albicans Candida* and rare yeasts in invasive infections. ^34,35^

The cloacal microbiota showed higher taxonomic diversity than the skin, supporting the gastrointestinal tract as a favorable ecological niche for several *Candida* and *Pichia* species. This distribution is biologically plausible, considering that the cloacal environment provides humidity, organic substrates, and nutrient availability that facilitate yeast colonization and persistence. ^29,36^

From a One Health perspective, the detection of multiple clinically important yeasts in captive reptiles deserves particular attention. Species such as the *C. parapsilosis* complex, *P. kudriavzevii, C. lusitaniae*, and *T. asahii* are recognized as opportunistic pathogens in human medicine and have been increasingly implicated in nosocomial infections and antifungal-resistant phenotypes. ^35,37,38^*Pichia kudriavzevii* (formerly *Candida krusei*) is included in the WHO fungal priority list due to its intrinsic resistance to FCZ and its clinical mortality rates. ^9,39^ Moreover, genomic studies have demonstrated limited distinction between environmental and clinical isolates of this species, reinforcing concerns regarding its environmental dissemination. ^40^ Similarly, *T. asahii* has emerged as an important pathogen in reptiles and has been associated with fatal systemic infections in captive lizards under stress conditions. ^41^ While fungal growth at 37°C was not explicitly evaluated in this study, this phenotypic trait represents a critical component for assessing the ability of environmental or wildlife-associated yeasts to adapt to and colonize mammalian hosts. Future surveillance studies incorporating thermal tolerance assays at human body temperature will be essential to precisely define the zoonotic potential and transmission dynamics of these reptile-associated mycobiota. Although colonization does not necessarily imply infection or zoonotic transmission, the presence of these fungi in captive reptiles highlights their potential epidemiological relevance, especially in environments characterized by frequent human-animal interaction.

A major strength of this study was the interpretation of antifungal susceptibility according to CLSI criteria, using CBPs and ECVs whenever available. This approach allowed a more cautious interpretation of reduced susceptibility patterns, particularly among uncommon or emerging yeast species for which standardized criteria remain limited. Crucially, a comparative analysis with free-living populations reveals a stark contrast in antifungal resistance dynamics: fungal isolates recovered from wild lizards exhibit 100% susceptibility to all tested antifungal agents, showing a complete absence of drug resistance phenomena ^8^ In contrast, our findings in captive lizards reveal prominent phenotypes with high MICs. Overall, antifungal susceptibility testing revealed marked phenotypic heterogeneity, with broad MICs distribution across all antifungal classes tested. Such variability is increasingly recognized among non-*C. albicans* and environmental yeasts and reflects the growing complexity of antifungal resistance epidemiology. ^42,43^

Reduced susceptibility to azoles was particularly evident in several isolates. Environmental exposure to azole compounds, including agricultural contamination, has been proposed as an important selective pressure contributing to the emergence of resistant fungal populations. ^11,44^ In this context, the detection of isolates exhibiting elevated MICs in animals without documented antifungal exposure may represent an early indicator of selective pressures acting within captive ecosystems.

Particularly concerning was the high frequency of isolates with MICs above thresholds for PSZ and AMB. Posaconazole resistance has increasingly been reported in clinical settings over the last decade, also with respect to the other azoles. ^45^ Considering the recognized activity of PSZ against *C. auris* ^46^ the recovery of environmental isolates with elevated MICs deserves attention. Likewise, although AMB resistance remains relatively uncommon in several *Candida* species, the recovery of isolates with reduced susceptibility from untreated animals suggests the need for further genetic and genomic investigations to clarify possible resistance mechanisms and environmental selective pressures. ^47,48^

In contrast, resistance or NWT phenotypes associated with AND remained less frequent. Nevertheless, the detection of elevated MICs in species such as *A. kalrae* and *M. caribbica*, together with the recovery of intrinsically echinocandin-resistant species such as *T. asahii* emphasizes the importance of species-level identification and careful interpretation of susceptibility results in uncommon yeasts.^33^ The broad MIC distributions observed for *A. kalrae* further support the hypothesis of heterogeneous susceptibility patterns rather than a single uniform phenotype, consistent with previous clinical reports describing variable antifungal responses in this species.^27^ From an epidemiological perspective, the high prevalence of *A. kalrae* in this cohort, coupled with these complex susceptibility profiles in a context lacking ongoing or targeted clinical antifungal treatments, provides robust support for the One Health framework regarding environmental resistance reservoirs. This prominent widespread colonization suggests that captive wildlife and their managed enclosures can act as significant, non-clinical hubs where opportunistic fungi with reduced drug susceptibility can persist and evolve, driven potentially by indirect environmental selective pressures rather than direct veterinary pharmaceutical intervention.

Interestingly, *M. caribbica* exhibited the highest MIC levels observed among the species analyzed.

Although closely related to *M. guilliermondii*, both species differed significantly in their susceptibility profiles. ^49^ If confirmed in larger studies, these findings may have important clinical implications considering the emerging role of the *Meyerozyma* complex as an opportunistic pathogen group. ^50^ Similarly, *M. guilliermondii* and *C. lusitaniae* showed both WT and NWT phenotypes, supporting previous evidence of emerging resistance to azoles and AMB in these species. ^28,51^ Notably, *C. lusitaniae* has been associated with reduced susceptibility to AMB in clinical settings. ^11,51^

Zoological institutions may occasionally rely on empirical antifungal treatments in reptiles, particularly in cases of dermatitis or systemic mycoses. ^52^ Although causal relationships cannot be established in this cross-sectional study, repeated or suboptimal antifungal exposure may contribute to the selection and persistence of less susceptible fungal populations causing a possible dissemination of fungal diversity with non-wild resistance patterns. Although causality cannot be established in the present cross-sectional study, our findings underscore the value of periodic surveillance programs integrating mycological and susceptibility data.

Nevertheless, some methodological limitations should be acknowledged when interpreting species-level identification and the corresponding resistance profiles. Although the complete concordance between MALDI-TOF MS and ITS sequencing provides strong support for the reliability of the species assignments obtained in this study, it does not eliminate the inherent limitations of single-locus identification. While the ITS region is the accepted primary fungal barcode, additional loci such as the LSU (D1/D2) region or the TEF1-α region may provide greater taxonomic resolution for certain phylogenetically complex yeast groups. Consequently, some isolates belonging to closely related species complexes could potentially have been inaccurately assigned to species with a lower degree of confidence than would be achieved using a multilocus approach. This could result in minor changes in the frequency of the species reported. Nevertheless, this limitation is unlikely to affect the overall diversity patterns observed, the predominance of the major yeast genera recovered, or the main ecological findings of the study. Similarly, antifungal susceptibility testing was performed independently of taxonomic assignment, so the reported MIC values reflect the phenotypes of the recovered isolates. However, caution should also be exercised when extrapolating species-specific susceptibility profiles within phylogenetically complex taxa.

We consider that these limitations do not substantially alter the ecological or One Health implications of our findings. Future studies incorporating multilocus sequencing will undoubtedly improve taxonomic resolution and further refine the epidemiological significance of these isolates.

Concluding, this study demonstrates that captive lizards harbor diverse yeast communities and may act as asymptomatic reservoirs of clinically relevant opportunistic yeasts. The predominance of *A. kalrae* and the wide fungal diversity observed provide new insights into the ecology of reptile-associated mycobiota under captive conditions. In addition, antifungal susceptibility profiles revealed marked heterogeneity among species, including variable azole activity, limited echinocandin efficacy, and elevated AMB MIC values in several taxa. From a One Health perspective, the detection of medically important species and heterogeneous antifungal susceptibility profiles highlights the potential epidemiological relevance of captive reptiles in the circulation of opportunistic and potentially resistant yeasts and calls for cautious interpretation of antifungal susceptibility in uncommon yeasts, particularly in the absence of established CBPs. Although the observed findings do not indicate an immediate public health threat, they reinforce the importance of periodic microbiological surveillance, prudent antifungal use, and targeted hygiene measures in zoological settings. Overall, these findings contribute to the growing evidence supporting reptiles as potential environmental reservoirs of opportunistic fungi and emphasize the need to include captive wildlife in broader fungal surveillance strategies.

## Data Availability

All data are available within the article.
